# Levels of Genetic Variants Among Symptomatic *Blastocystis* Subtypes and their Relationship to Mucosal Immune Surveillance in the Precancerous Colons of Experimentally Infected Rats

**DOI:** 10.1007/s11686-022-00628-z

**Published:** 2022-11-15

**Authors:** Eman M. Hussein, Muhammad A. A. Muhammad, Abdalla M. Hussein, Sherine M. Elzagawy, Wafaa M. Zaki, Ashraf G. Temsah, Mohamed S. Badr, Maha M. Alabbassy

**Affiliations:** 1 Medical Parasitology Department, Faculty of Medicine, Suez Canal University, Ismailia, 41522 Egypt; 2 Pathology Department, Faculty of Medicine, Suez Canal, University, Ismailia, 41522 Egypt; 3 Bio-Physics Department, Faculty of Science, Al-Azhar University, Cairo, 11652 Egypt; 4 Medical Parasitology Department, Faculty of Medicine, Damietta Branch, AL Azhar University, Damietta, Egypt; 5 Medical Genetic Centre, Molecular Biology Department, Faculty of Medicine, Ain Shams University, Cairo, Egypt

**Keywords:** *Blastocystis*, Intrasubtype, Immune surveillance, MUC2, Precancerous, Polyps

## Abstract

**Purpose:**

The relationship between the genetic diversity of *Blastocystis* and immune surveillance in precancerous colons with blastocystosis is still under investigation. This study aimed to identify the genetic *Blastocystis* variants among 54 symptomatic human isolates and their relationship to mucosal immune surveillance in the precancerous polyps of experimentally infected rats.

**Methods:**

Polymerase chain reaction and high-resolution melting (PCR/HRM) curves discriminated human symptomatic *Blastocystis* isolates into subtypes (STs)/intrasubtypes, which were orally administered to rats to induce experimental infection. Then, the mucosal immune responses of the infected colons were evaluated in relation to polyp formation through immunostaining to identify mucus MUC2 and determine mucosal immune cell (goblet, lymphocyte and mast) counts, secretory IgA levels and parasitic intestinal invasion.

**Results:**

ST1, ST3, and ST4 were found in 18.5% (10/54), 54.7% (29/54), and 27.8% (15/54) of the samples, respectively. Then, the HRM curve discriminated ST3 into the wild, mutant, and heterozygous [17/54 (31.5%), 5/54 (9.3%), and 7/54 (12.9%)] intrasubtypes. ST1 and ST4 had no genetic variations. Precancerous polyps were detected in the colons of 40.5% of the infected rats. ST1 constituted 14.7% of these cases, while the wild, mutant, and heterozygous intrasubtypes of ST3 showed polyps in 12.9%, 5.5%, and 5.5% of cases, respectively. Only 1.9% of the polyps were related to ST4. MUC2 showed weak immunostaining in 44.5% of the infected colons, and 38.9% were polyp inducers. Low goblet cell numbers and high interepithelial lymphocyte counts were significantly associated with polyp formation, particularly with ST1 and wild ST3. Among the polyp inducers, high numbers of mast cells were detected in wild ST3 and ST4, while a low number was found with heterozygous ST3. The level of secretory IgA was low in polyp-inducing STs. Most of the results were statistically significant.

**Conclusion:**

Immunosurveillance showed a potential relationship between ST1 and the ST3 intrasubtypes and precancerous polyps. This relationship may provide insight into the prevention and/or development of new immunotherapeutic strategies to combat colorectal cancer.

## Introduction

*Blastocystis*, the most common eukaryotic organism worldwide, inhabits the intestinal tract of approximately two billion humans and a wide range of animals [60]. Indeed, *Blastocystis* presents in both patients and healthy individuals, so its role in disease development has yet to be established [20]. Although up to 25 genetic subtypes (STs) of *Blastocystis* have been found in birds and mammals based on the small-subunit (SSU) rRNA gene sequences, only STs 1–9 and 12 have been detected in humans [42, 65]. STs 1–4 constitute 90% of human *Blastocystis* [4]. Recently, phylogenetic and sequencing studies added intrasubtype and intersubtype variants, providing wide genetic diversity of *Blastocystis* [64]. High-resolution melting (HRM) curves are a novel post-polymerase chain reaction (PCR) method that analyses genetic variations via single nucleotide polymorphisms, mutations and methylations in PCR amplicons based on the GC/AT ratio, length, and distribution [27]. The PCR/HRM curve has differentiated *Blastocystis* STs into wild, mutant, and heterozygous variants [70]. These intra- and intersubtype variabilities exhibit remarkable differences in the outcome of *Blastocystis* infection [73].

The pathogenicity of *Blastocystis* depends on the induction of intestinal epithelial cell apoptosis [65]. In addition, *Blastocystis* modulates host immune responses by upregulating or downregulating certain inflammatory cytokines [52]. Moreover, *Blastocystis* symptomatic isolates exhibit high cysteine protease levels that facilitate the downregulation of epithelial antiparasitic nitric oxide formation [16, 44]. Interestingly, solubilized antigens of *Blastocystis* induce the *in vitro* proliferation of HCT116 human colorectal carcinoma cells [14, 15]. Nevertheless, Chen *et al*. [17] showed that 17.3% of *Blastocystis-*infected patients had large intestinal polyps (colorectal adenoma). Likewise, the blastocystosis prevalence rate is 34% among patients with colorectal adenoma [63]. Colorectal adenoma carries a high risk of developing into colorectal cancer (CRC) as a premalignant lesion [10]. Recently, *Blastocystis* parasites were identified in 12.15% of European CRC patients versus 2.42% of controls [67]. Notably, these precancerous polyps have been found in rats experimentally infected with *Blastocystis* ST1 and ST3 [1, 28]. In addition, *Blastocystis* obtained from CRC patients had different tissue proliferation and invasion capabilities in experimentally infected mice [2].

Up to 20% of cancer cases worldwide are associated with infection (a major driver of chronic inflammation), which plays an important role as a tumor promoter during immunosurveillance [23]. Mucus is excreted by goblet and epithelial cells and is an important part of the gastrointestinal tract (GIT) immunity [30]. Goblet cells synthesize MUC2, which is the most protective mucin in the GIT [49]. Patients with CRC have shown weak expression of MUC2 [35]. In addition to goblet cells and their mucins, cytotoxic intraepithelial lymphocytes (IELs) are also involved in mucosal immunosurveillance and have significant impacts in CRC [21]. Additionally, mast cells are an integral feature of the tumor microenvironment [29]. ST1 and ST3 facilitate mast cell activation during blastocystosis [11, 41]. Furthermore, fecal secretory immune globulin A (sIgA), a potential marker for CRC screening and early detection [13], is predominant during blastocystosis [39, 59]. However, the immunological changes that occur during blastocystosis that lead to carcinogenesis are not well established. Therefore, in the present study, PCR/HRM curve analysis was used to explore the levels of intrasubtype *Blastocystis* variants from Egyptian human symptomatic isolates and their relationship to immune surveillance of the precancerous colon in rats experimentally infected with *Blastocystis*.

## Materials and Methods

### The Source of the *Blastocystis* Isolates

Stool specimens from 350 patients with gastrointestinal symptoms attending the gastroenterology and tropical medicine outpatient clinics of Suez Canal University Hospitals were examined to diagnose *Blastocystis* infection. There were 138 stool samples containing *Blastocystis*. Among them, 63 samples with *Blastocystis* as the only intestinal pathogen were selected. *Blastocystis* isolates propagated by stool culture were subjected to ST identification by conventional PCR. Subsequently, 54 *Blastocystis* isolates were involved in PCR/HRM and infection experiments, while 9 samples with mixed ST infection were excluded.

### Sample Selection

Stool samples collected from the GIT symptomatic patients were processed and examined according to Garcia [24]**.** Immediately after acquisition, the samples were subjected to direct examination by the naked eye. Wet smears with saline stained with Lugol’s iodine were created for all of the human stool samples to identify the *Blastocystis* diagnostic stage and other intestinal parasitic infections with a light microscope. Then, concentration analyses were performed to exclude mixed parasitic infections that may be responsible for the GIT symptoms that were not detected in the wet mounts. Four grams of stool was mixed thoroughly with 10% formalin and concentrated via formalin ethyl acetate sedimentation methods. Two thin smears from each concentrated stool sample were examined by light microscopy (one was stained with Lugol’s iodine, and the other was unstained). Another thin smear from each concentrated sample was subjected to staining with modified acid-fast trichrome and examined by light microscopy to exclude samples containing *Cryptosporidium*, *Cyclospora cayetanensis*, *Cystoisospora*, and Microsporidium. Moreover, samples mixed with common intestinal bacteria were excluded by applying Salmonella-Shigella and MacConkey agar for stool cultures [32]. Additionally, a *Dientamoeba fragilis* stool culture using modified Robinson’s medium was performed [24], and a Rapid immunochromatographic Adeno-Rotavirus Rapid dipstick test (DiaSys, USA) was used for the detection of adenovirus and rotavirus antigens according to the manufacturer’s instructions. There were only 63 patients with *Blastocystis* as the only intestinal pathogen present in their stools, and these samples were selected for further study. Approximately 50 mg of each stool sample positive for *Blastocystis* was inoculated in 10 ml of sterilized modified Jones medium containing 10% heat-inactivated (56 °C for 30 min) horse serum, 100 μg/ml streptomycin, and 100 IU/ml penicillin. For *Blastocystis* propagation, the inoculated medium was incubated at 37 °C for 4 days [31].

### Molecular Characterization of Human *Blastocystis:*

#### DNA Extraction

*Blastocystis* was isolated from modified Jones culture by centrifugation at 400 × g for 10 min. After discarding the supernatant, the pellet was suspended in phosphate-buffered saline (PBS; pH 7.4). This suspension was overlaid onto a Ficoll-Paque column and centrifuged at 2000 × g for 10 min. *Blastocystis* forms separated into a band approximately 1 cm from the surface. This layer was collected and resuspended in 8 ml of PBS and centrifuged at 500 × g for 5 min; this step was repeated six times. The resultant pellet was resuspended in 1 ml of PBS and centrifuged at 500 × g for 5 min [46, 48], and the final resultant pellet was stored at − 20 °C until required for DNA extraction. DNA extraction was performed from the pellets of concentrated samples after culture propagation using a QIAamp DNA Mini Kit (Qiagen, Germany) according to the manufacturer's protocol.

#### PCR/HRM

PCR/HRM was performed based on the SSU rRNA gene using seven STS (Table 1) primers [75]. PCR was performed in a total volume of 50 µL containing 20 μl of template DNA, 10 mM Tris–HCl (pH 9.0), 50 mM KCl, 0.1 Triton X-100, 2 mM MgCl_2_, 200 μM each of dNTPs, dCTP, dGTP, and dTTP, 0.2 μM each primer, and 1.25 U of Taq DNA. The amplification round began with the initial activation of the HotStar Taq DNA polymerase at 95 °C for 15 min. Then, the PCR procedure consisted of one cycle of denaturing at 94 °C for 3 min, 30 cycles of annealing at 59 °C for 3 s, extending at 72 °C for 60 s, and denaturing at 94 °C for 30 s, followed by an additional elongation cycle for 5 min at 72 °C. Ten microlitres of the PCR product in an agarose slot was electrophoresed on a 2.5% gel and photographed with UV transillumination. The DNA marker and the control samples (positive and negative) obtained from our previous study were used as a guide in each electrophoresis run [28]. Nine samples with mixed infection subtypes were excluded. In each real-time PCR well, SYBR green 1 dye (Roche) (1:40,000 final dilution) and 1.5 U of Fast Star *Taq* DNA polymerase (Applied Biosystems) in a total volume of 20 µL of conventional PCR products was used. All of the reactions were performed in triplicate for each (isolate) ST. Immediately upon the completion of amplification, a melting program was followed in a thermal cycler (LightCycler® 480, Roche, Molecular system). The program was as follows: 95 °C/min, 40 °C/1 min, and 65 °C/1 s then 95 °C with different ramp increments from 0.2 °C/s, followed by 40 °C/30 s (cooling phase). The changes in fluorescence were recorded and plotted as a function of the change in temperature (*d*F/*d*T) [27]. HRM curve analysis was performed using LightCycler® 480 gene scanning software. Positive and negative controls were included in every run. For intrasubtype genotyping, the fluorescence intensity raw data (melting curves) were normalized by selecting the linear regions before and after the melting transition to define two lines for each curve: an upper fluorescence line (100%) and a lower baseline (0%) before and after the melting transition of each sample. The acquisition fluorescence of each sample/ST was calculated as the percentage of fluorescence between the top and bottom baseline at each acquisition temperature. Different intrasubtypes were discriminated by plotting the fluorescence between the normalized melting curves. Normalization and background subtraction were first performed to identify the diagnostic features by fitting the background surrounding the HRM transition of interest to an exponential curve. The normalized HRM curve temperature was overlaid to eliminate slight temperature errors between wells or runs. Then, different plots of these normalized HRM curve temperature-overlaid curves were obtained by deducting the fluorescence difference of each curve from the average common (wild) curve at all temperatures [26]. The HRM profile with a plot that was interpreted by software to be different from the averaged wild-type curve was considered to have minimal or frank nucleotide changes (mutation or heterozygous variants). The reproducibility of the HRM was investigating by performing the experiments in triplicate on all of the samples yielding either frank or minimal variations in the normalized HRM curve. To assess the reproducibility of the methods, the second round of HRM was performed using negative and positive controls. Minimal variation was detected as the minor variance for the wild-type (3%).

**Table 1:** STS primers according to the method of Yoshikawa *et al*. [75]

	GenBank accession no	STS	Base pair	Primer	Sequences (3–5)
Subtype 1	AF166086	SB83	351	Forward Reverse	GAAGGACTCTCTGACGATGA GTCCAAATGAAAGGCAGC
Subtype 2	AF166087	SB155	650	Forward Reverse	GTCCAAATGAAAGGCAGC GTCCAAATGAAAGGCAGC
Subtype 3	AF166088	SB227	526	Forward Reverse	TAGGATTTGGTGTTTGGA GA TTAGAAGTGAAGGAGATGGAAG
Subtype 4	AF166091	SB332	338	Forward Reverse	GCATCCAGACTACTATCAACATT CCATTTTCAGACAACCACTTA
Subtype 5	AY048752	SB340	704	Forward Reverse	TGTTCTTGTGTCTTCTCAGCTC TGTTCTTGTGTCTTCTCAGCTC
Subtype 6	AY048751	SB336	317	Forward Reverse	GTGGGT AGAGGAAGGAAAACA AGAACAAGTCGATGAAGTGAGAT
Subtype 7	AY048750)	SB337	487	Forward Reverse	GTCTTTCCCTGTCTATTCTGCA AATTCGGTCTGCTTCTTCTG

#### Experimental Study

Four-week-old male western rats (Sprague–Dawley) weighing 150–200 g were obtained from the experimental house [three per isolate (subtype or intrasubtype) with 6 in the healthy control group] at the Faculty of Medicine, Suez Canal University. The rats were housed under standard animal housing conditions. The infection doses were 7 × 10^4^ *Blastocystis* cysts from 4-day-old-axenic culture according to Hussein *et al*. [28]. Feces from all rats were examined microscopically on Days 3 and 7 postinoculation by wet mount with Lugol's iodine staining or culture to confirm the presence of *Blastocystis* forms. At 8 weeks after the confirmation of infection, the rats were sacrificed for histopathological examination. Tissue samples from the walls of the caeca and proximal colons of the sacrificed animals (infected and controls) were fixed in 10% neutral buffered formalin for haematoxylin and eosin staining [8].

#### Evaluation of Mucosal Immune Surveillance of the Colon

##### Immunohistochemistry

To identify tissue MUC2, formalin-fixed, paraffin-embedded sections from the caeca and colons of infected rats and control rats were stained using monoclonal antibodies directed against MUC2 (clone Ccp58, dilution 1:100; Novocastra) glycoproteins. Specimens were deparaffinized in xylene and rehydrated in a graded ethanol series. Antigen retrieval was performed by steam heating slides in citrate buffer (pH 6.0) in a steamer (Black and Decker, Shelton, CT) for 20 min. Staining was performed using an automated immunostainer (DAKO, Carpinteria, CA), followed by antibody detection using the DAKO EnVision + System and 3,3'-diaminobenzidine as the chromogen. The slides were counterstained with haematoxylin, and coverslips were applied. Appropriate positive and negative tissue control samples were used. Sections were washed twice and fixed in PBS containing 2% (w/v) formaldehyde. Then, the sections were incubated overnight with a 1000 × dilution of the primary antibody against MUC2 (Sigma; 1:1000 in PBS) at 4 °C. After washing the sections twice with PBS, the specimens were incubated for 1 h with the secondary antibody, followed by another round of washing. Negative control and positive control slides were used. The identification of MUC2 was performed semiquantitatively with a scoring system [6] as follows: the score was 0 when negative or no staining was observed; 1, when less than 5% of the cells were stained; 2, when 5–25% of cells were stained; 3, when between 25 and 35% of the cells were stained; and 4, when more than 35% of the cells were stained. Scores of 3 and 4 indicate strong reactions, while scores of 0–2 indicate weak reactions.

##### Light Microscopy Tissue Examination

According to the methods used by Yunus *et al*. [77] for Periodic acid–Schiff (PAS) staining and of goblet cells counting, a sample approximately 2 cm in length was taken from the middle portion of each caecum and colon. After washing the segments twice with saline, the specimens were fixed in Carnoy’s fixative for 2 days. The fixed tissues were embedded in paraffin and sectioned at a thickness of approximately 5 μm. Briefly, the samples were oxidized with 1% periodic acid (Sigma‒Aldrich) for 5 min and reacted with Schiff reagent to produce a colored end product. The samples were counterstained with haematoxylin (Sigma‒Aldrich). Goblet cell numbers were counted per 10 villus-crypt units (VCUs). IEL staining and counting were performed one cm from the caecum and colon [8]. The tissues were fixed in Carnoy’s fixative for 24 h then sectioned at a thickness of 5 μm after dehydration and embedding in wax. After that, the sections were stained with 2% Giemsa solution for 20 min. According to their relationship to epithelial cell nuclei, the apical, middle, and basal IELs were counted per 10 VCUs**.** For mast cell counting, sections from the caecum and colon were prepared as described above for IEL counting. At pH 0.3, the sections were stained with toluidine blue and counterstained with weak eosin. The granulated mast cells were counted per 10 VCUs [66]. Goblet cells, IELs, and mast cells were counted three times for each isolate and recorded as the mean ± standard deviation (S.D.).

##### Transmission Electron Microscopy (TEM) Examination of Tissues

Tissue samples were obtained from the caeca and colons of rats infected with each subtype/intrasubtype with and without polyp formation [78]. Tissue samples were washed with 0.2 M sodium cacodylate buffer. Then, the specimens were incubated overnight at 4 °C with a mixture of 4% glutaraldehyde. After washing three times with 0.2 M sodium cacodylate buffer, the samples were postfixed for 1 h with 1% OsO4 in 0.2 M sodium cacodylate buffer. Then, the specimens were washed three times (10 min) in 0.2 M sodium cacodylate buffer and dehydrated in graded ethanol and acetone solutions. Next, the samples were infiltrated with acetone and EPON resin mixture (2:1) for 1 h, with acetone and EPON resin mixture (1:1) for 1 h, and with acetone and EPON resin mixture (1:2) for 1 h. Subsequently, specimens were embedded in resin overnight at RT and cured for 2 days in a 60 °C oven. Thin Sects. (70 nm) were cut using a UC6 ultramicrotome (Leica, Wetzlar, Germany) and stained with uranyl acetate and lead citrate. Carbone was evaporated using a CE6500 unit. Specimen sections were observed at 80 kV (JEOL JEM.1400 TEM). Electron microscopy preparations and examinations were performed by the Electron Microscopy Unit of Al-Azhar University, Cairo. Egypt.

##### Measurement of sIgA in the Different Groups of *Blastocystis*-Infected Rats

From the large intestinal walls of sacrificed rats (infected and control), tissue samples were rinsed with ice-cold PBS (pH 7.4) to remove blood. After weighing the tissue specimens, each sample was disaggregated by ultrasonication (20 kHz, 1 mA) for 30 s for six cycles in an ice bath with 30 s intervals. Thereafter, centrifugation of the homogenate was performed at 5,000 × g for 5 min at 4 °C, and the supernatant was aliquoted. The supernatant with *s*IgA was adjusted spectrophotometrically to a final concentration of 5 mg of protein per ml. The concentration of sIgA in these supernatants was assayed with two-site sandwich enzyme-like immunosorbent assays (ELISAs) using ELISA kits according to the manufacturer’s instructions (Wuhan Elabscience Biotechnology Co., Ltd) [61]. The optical densities (ODs) of sIgA were measured three times for each isolate and were recorded as the mean ± standard deviation (S.D.).

### Statistical Analysis

Statistical significance was determined using chi-square/Fisher exact tests**.** Unpaired Student’s t test was used to compare the means ± S.D. of the different immune cell numbers and the level of sIgA between the different subtypes/intrasubtypes in both the colons with and without polyps to calculate the degrees of freedom. A P value ≤ 0.05 was defined as statistically significant.

## Results

**Fig. 1:**
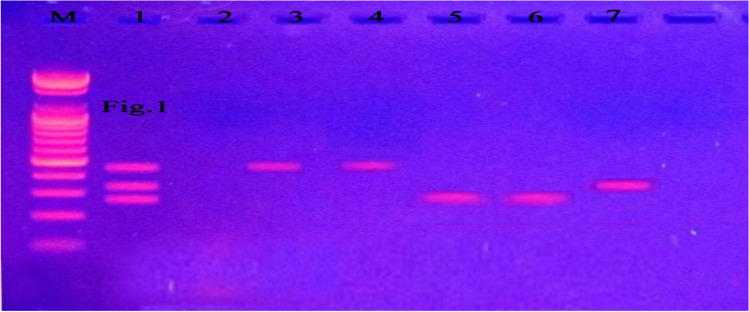
Stained agarose gel with PCR products amplifying DNA extracted from some stool samples (as examples) of Blastocystis symptomatic patients shows three subtypes. M is the ladder. Lane 1&2 are the control + Ve and -Ve, respectively. Lane 3 & 4 represent ST3 at 526 bp. Lanes 5 &6 show ST4 at 338 bp. Lane 7 demonstrates ST1 at 351 bp

**Fig. 2:**
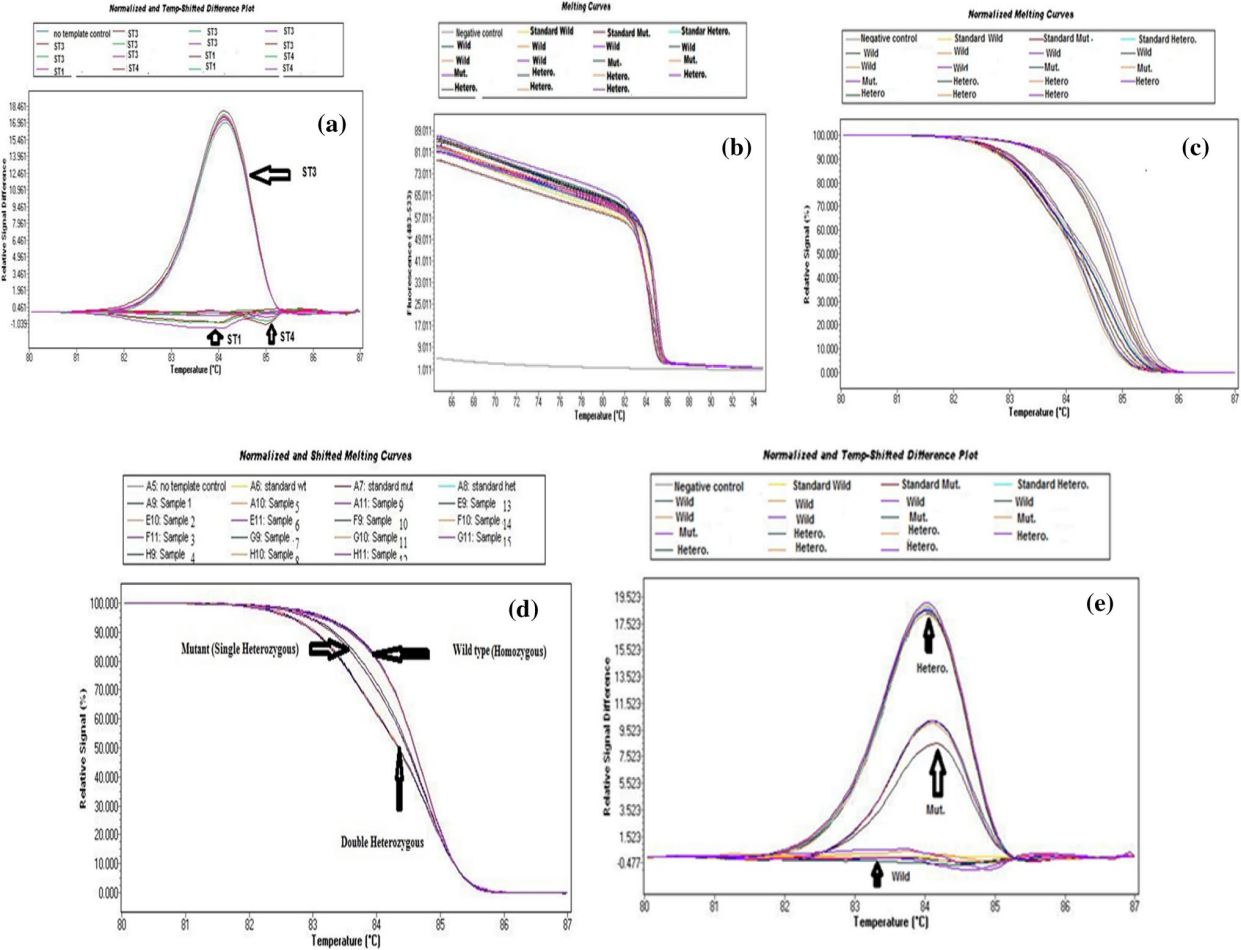
**a–c**. **a** shows the Common/Variant’s analysis mode places all symptomatic isolates into either the common group (the largest group of samples with similar melting curve shape) or into the variants group. **b** demonstrated the melting curve analysis of subtype 3 with the largest group of samples with similar melting curve shapes and the variants group. **c** shows normalized melting curve analysis discriminates ST 3 into either common group and variants group. **d–e**; **d** shows the Common/Variants analysis of ST 3 using normalized and shifted melting curves discriminates them into the wild, mutant heterozygous and double heterozygous. **e** confirms the result by Normalized and Temp-Shifted Difference Plot analysis

**Fig. 3:**
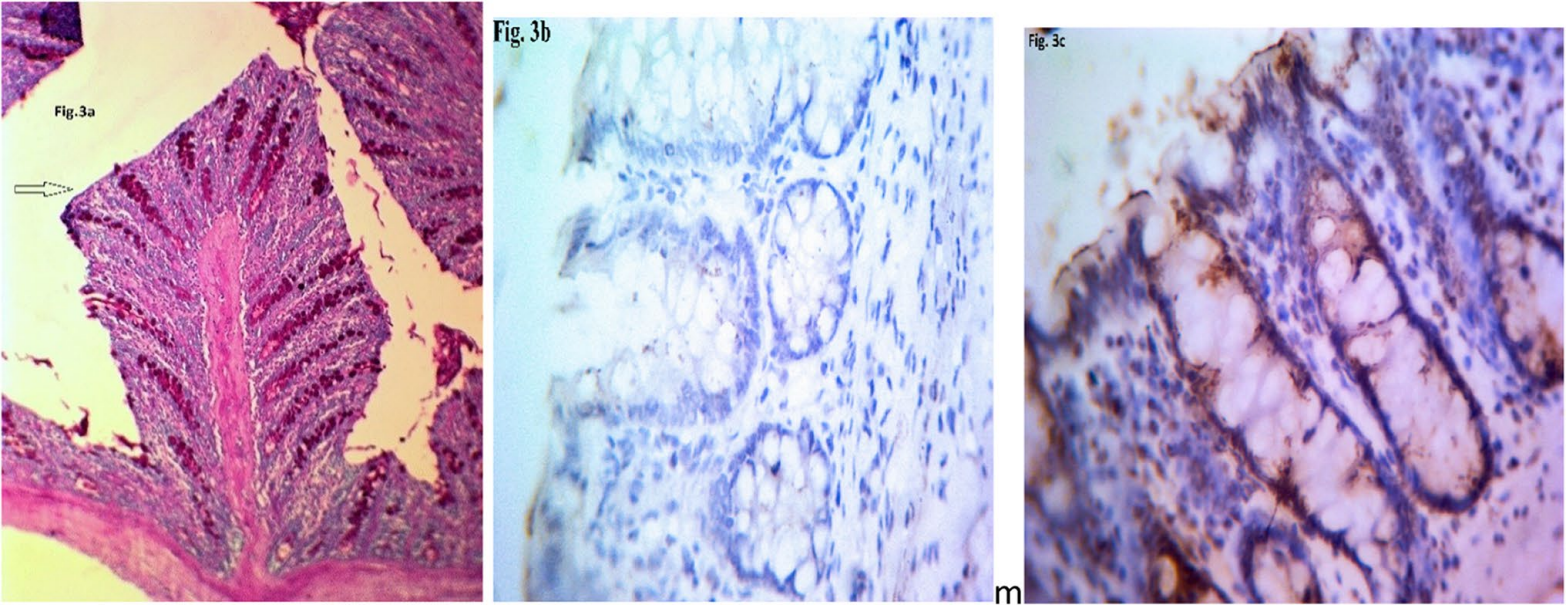
**a–c**: shows Intestinal polyps stained with PAS (**a**), mucosa shows MUC2 weak immune stained (**ab**) and intestinal mucosa shows MUC2 strong immune stain with brownish coloration X 400 (**a**)

In the present study, the distribution of *Blastocystis* STs among the 54 isolates obtained from the GIT symptomatic patients was 18.5% (10 isolates) ST1, 54.7% (29 isolates) ST3, and 27.8% (15 isolates) ST4 according to PCR analysis (Fig. 1). Regarding the level of genetic diversity shown by PCR/HRM, only ST3 demonstrated intrasubtype genetic variants, constituting 17/54 (31.5%) of wild isolates, 5/54 (9.3%) mutant and 7/54 (12.9%) heterozygous (Fig. 2a–e). ST1 and ST4 showed no intrasubtype genetic diversity. Regarding the induction of polyps by all STs, ST1 induced 8/54 (14.7%), the ST3 wild, mutant, and heterozygous variants induced polyps in 7/54, 3/54, and 3/54 (14.7%, 5.5%, and 5.5%) of the samples, respectively, while ST4 induced polyps in only 1/15 (1.9%) (Fig. 3a). These differences were statistically significant (Table 2).

**Table 2:** Frequencies of subtypes/intrasubtypes among *Blastocystis* gastrointestinal symptomatic isolates according to PCR/HRM with the presence or absence of polyps among experimentally infected rats

Polyps formation	ST1		Wild ST3		Mutant ST3		Hetero ST3		ST4		Total		*p* value
	No	%	No	%	No	%	No	%	No	%	No	%	
Polyps	8	14.7	7	12.9	3	5.5	3	5.5	1	1.9	22	40.5	0.006
No polyps	2	3.8	10	18.6	2	3.8	4	7.4	14	25.9	32	59.5	
Total	10	18.5	17	31.5	5	9.3	7	12.9	15	27.8	54	100	

The differences were statistically significant

Regarding MUC2 immunostaining reactivity, 24/54 (44.5%) of the isolates gave a weak staining reaction, while strong reactions were shown for 30/54 (55.5%) of the isolates. Precancerous polyps were induced with 21/54 (38.9%) of the isolates (Fig. 3b–c), eight (14.8%) of which were ST1, wild-type ST3 gave a weak reaction in 9/17 (16.8%) of the isolates (two did not induce polyps), the ST3 mutant with weak staining was detected in 2 (5%) of the isolates and the polyp inducer, and heterozygous ST3 gave a weak reaction in four (7.4%) of the isolates (one had no polyps). The ST4 isolate-inducing polyp gave a weak immunostaining reaction (1.9%). Otherwise, one mutant ST3 isolate gave a strong staining result and induced polyps in the colon. These differences were statistically significant (Table 3).

**Table 3:** Relation between infection with symptomatic *Blastocystis* subtypes/intrasubtypes and grades of MUC 2 immunostaining of colon of experimentally infected rats

MUC2 stain	ST1		Wild ST3		Mutant ST3		Hetero ST3		ST4		Total		*p* value
	No	%	No	%	No	%	No	%	No	%	No	%	
Weak	8	14.7	9*	16.7	2	3.8	4**	7.4	1	1.9	24	44.5	0.05
Strong	2	3.8	8	14.8	3***	5.5	3	5.5	14	25.9	30	55.5	
Total	10	18.5	17	31.5	5	9.3	7	12.9	15	27.8	54	100	

The differences were statistically significant

^*^Two isolates did not induce polyps

^**^One isolate did not induce polyps

^***^One isolate induced polyps

**Fig. 4:**
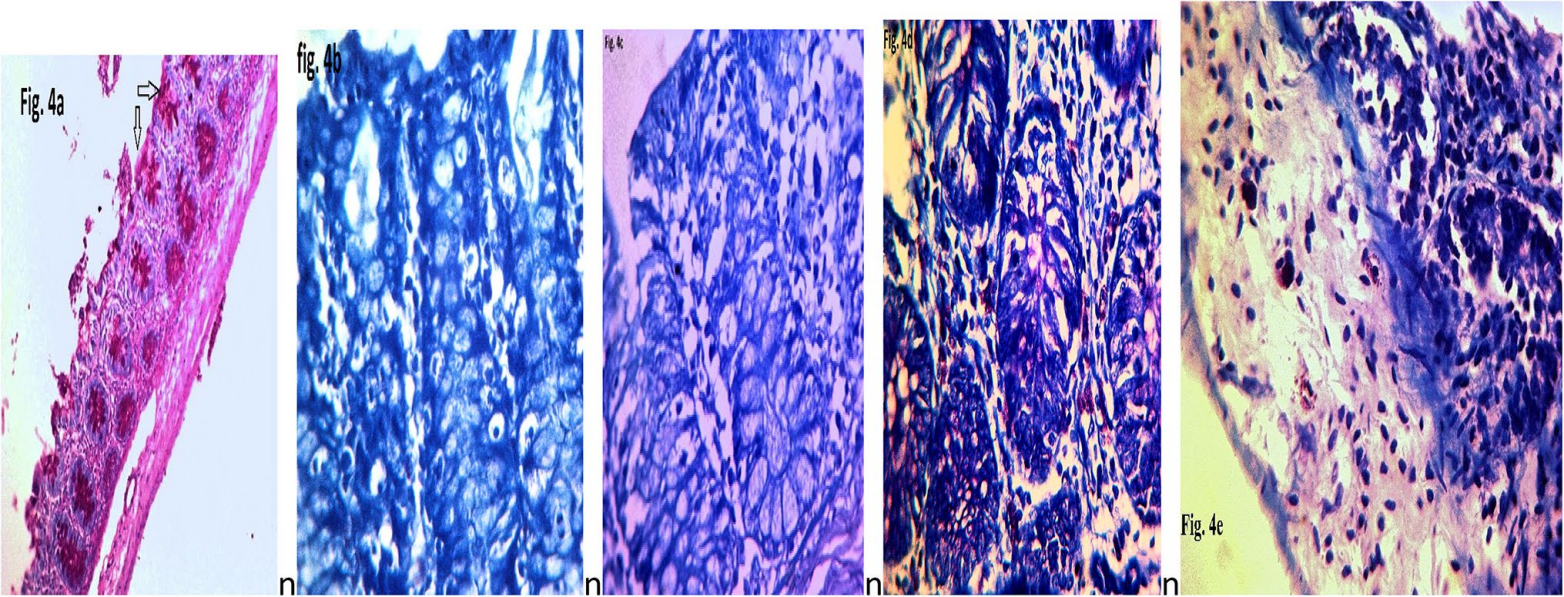
**a** shows PAS-stained goblet cells, **b** shows many lymphocytes stained with Giemsa in apical and middle parts, **c** shows few lymphocytes in the middle part. **d–e** many and few, respectively, mast cells stained with Toluidine blue X 400

The staining and counting of mucosal immune cells by light microscopy (Fig. 4a–e) revealed variations between polyp inducers and nonpolyp inducers (Table 4). The goblet cell count mean ± S.D.in all ST-induced polyps was less than that in the noninduced polyps. Among polyp inducers, ST1 gave the lowest count (40.47 ± 42.30), with a maximum count of 201 ± 5.0 from ST4. Among nonpolyp inducers, the counts were high and ranged from 205.50 ± 1.03 to 275 ± 5.0. The differences were statistically significant.

**Table 4:** Relation between infection with symptomatic *Blastocystis* subtypes/intrasubtypes and mucosal immune cells (Goblet, IELs, and Mast) counts on the colon of experimentally infected rats

Mucosal immune cells		ST1	Wild ST3	Mutant ST3	Hetero ST3	ST4
		Mean ± SD	Mean ± SD	Mean ± SD	Mean ± SD	Mean ± SD
Goblet	Polyp	40.67 ± 42.30	51.78 ± 39.39	146.67 ± 6.83	190.0 ± 5.0	201.0 ± 5.0
	No polyp	205.0 ± 5.0	252.50 ± 11.02	245.0 ± 5.0	275.0 ± 5.0	275.0 ± 5.0
	*p* value	0.0001	0.0001	0.05	0.05	0.05
IELs	Polyp	96.25 ± 4.33	88.33 ± 11.18	20.00 ± 3.16	10.50 ± 1.87	40.00 ± 5.00
	No polyp	17.33 ± 5.77	55.71 ± 4.50	7.00 ± 0.89	5.0 ± 1.00	20.00 ± 5.00
	*p* value	0.0001	0.001	0.05	0.05	0.05
Mast	Polyp	16.75 ± 14.37	34.00 ± 9.90	5.00 ± 0.89	1.00 ± 0.89	25.00 ± 2.00
	No polyp	4.0 ± 1.0	8.00 ± 0.89	2.50 ± 0.55	24.00 ± 0.89	8.00 ± 0.89
	*p* value	0.1*	0.001	0.001	0.0001	0.001

Among the control group, the Mean ± SD counts of goblet, IELs, and mast cells were 40 ± 1. 20, 3 ± 0. 20, and 1 ± 1.35, respectively**.** Almost the differences were statistically significant

^*^insignificant

Regarding the mean ± SD of the IELs, all STs/intrasubtypes that induced polyps showed high values in comparison to the colons without polyps and control. Among polyp inducers, moderate infiltrations (more than 100) were observed for ST1 (96.25 ± 4.33) and wild ST3 (88.33 ± 11.18), while heterozygous ST3 showed the lowest count (10.50 ± 1.87). Among the nonpolyp inducer STs/intrasubtypes, the counts ranged from 5.0 ± 1.0 to 55.71 ± 4.50. These differences were statistically significant. Mast cells showed variable numbers, as high counts were recorded among both polyp inducers and noninducers. Among the polyp inducers, the wild intrasubtype of ST3 showed the highest count (34.00 ± 9.90), while heterozygous ST3 showed the lowest (1.0 ± 0.89). Among the nonpolyp inducer STs, the counts ranged from 2.5 ± 0.55 to 24.00 ± 0.89. In the case of ST1, the count was 16.75 ± 14.37 among polyp inducers, while it was 4.0 ± 1.0 among the nonpolyp inducers. The differences were statistically significant with the exception of ST1.

**Fig. 5:**
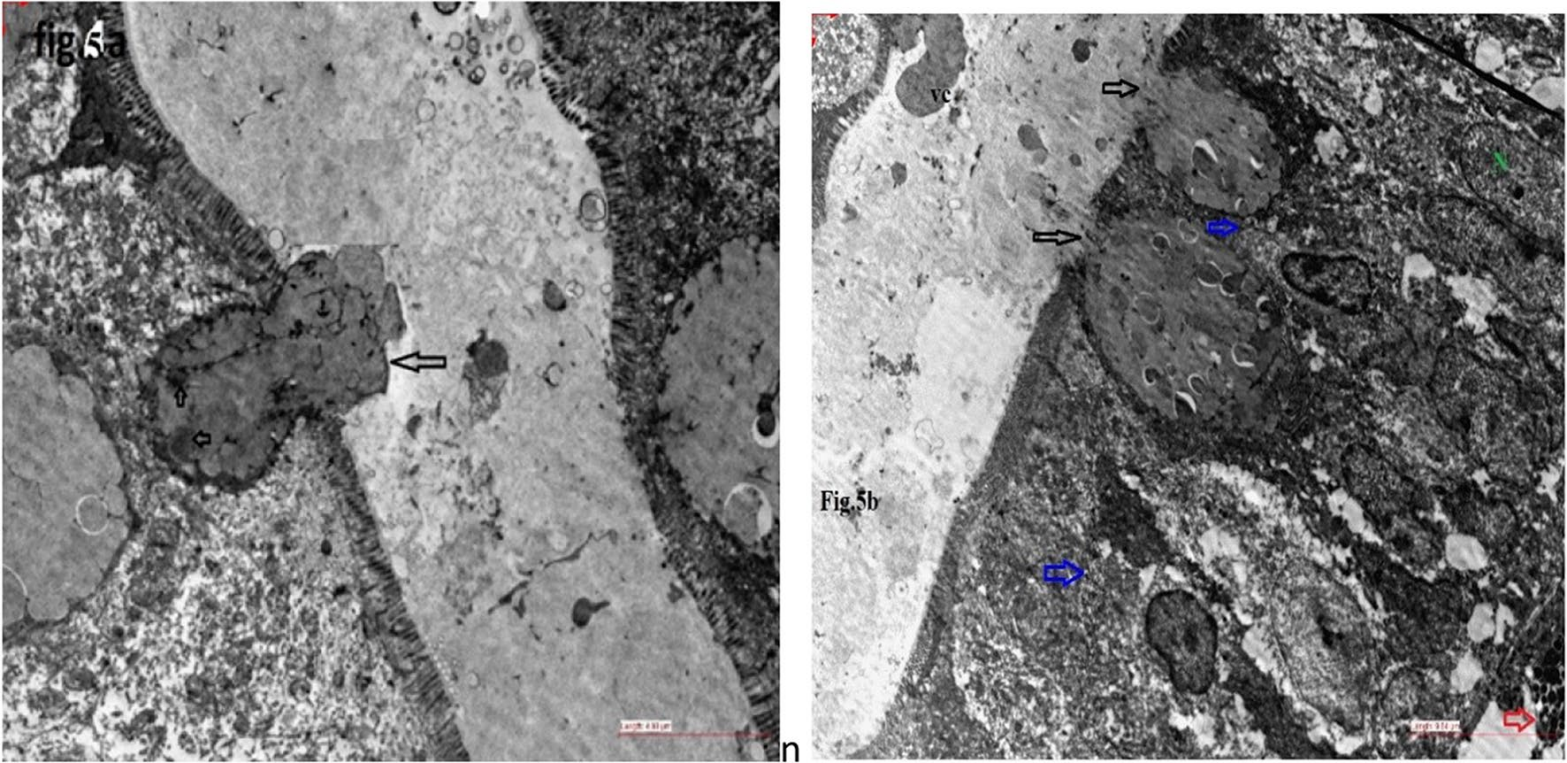
**a–b** shows TEM of the colon of experimentally infected rats with di nucleated (small arrows) parasite invading the mucosal layer (**a**). **b** shows activated Goblet cells (black arrow), IELs (blue arrows), mast cell (red arrow), nucleus (N) of epithelium and Blastocystis vacuolar form (VC) form

TEM examination showed that the parasite was located in the enteric cavity on the surface of the ileocecal and colonic mucosa. Partial destruction of the mucosal microvilli and the presence of activated goblet cells, IELs, mast cells and edema were observed. Vacuolar forms of *Blastocystis* invaded the mucosal layer of the colon (Fig. 5a–b). Regarding polyp formation, all ST-induced polyps showed invasion (100%), while only two nonpolyp inducer isolates (one ST1 and one wild ST3 intrasubtype) were invasive (8.6%).

In this study, the sIgA OD cut-off values for each ST/intrasubtype proved that the levels of sIgA among rats infected with ST/intersubtype-induced colon polyps were lower than that among rats infected with nonpolyp inducers (Table 5). Among the polyp-containing colons, the counts ranged from 0.420 ± 0.19 to 1.986 ± 2.123, while in the colons without polyps, the counts ranged from 2.006 ± 0.008 to 3.486 ± 2.123, and these differences were statistically significant,

**Table 5:** Mean ± SD of sIgA ODs cut of values among infected rats in relation to subtypes/intrasubtypes frequencies and polyps formation

Colon lesions	ST1	Wild ST3	Mutant ST3	Hetero ST3	ST4
	Mean ± SD	Mean ± SD	Mean ± SD	Mean ± SD	Mean ± SD
Polyps	0.420 ± 0.19	1.122 ± 0.007	1.192 ± 0.008	0.926 ± 0.19	1.986 ± 2.123
No polyps	2.617 ± 1.121	3.486 ± 2.123	2.006 ± 0.008	2.010 ± 0.09	2.1532 ± 0.025
*p* value	0.0001	0.001	0.001	0.005	0.05

The cut of value for controls was 0.210 ± 0.09. The differences were statistically significant

## Discussion

*Blastocystis* ST1 and ST3 constitute a major risk factor for the occurrence of CRC (46; 34]. In a study of CRC patients, ST3 was detected in 75% of them, followed by ST1 in 16.7% and ST2 in 8.3% [67]. However, few studies have been carried out to assess the role of *Blastocystis* genetic variations in immune surveillance associated with the presence of precancer in the colon.

In the current study, conventional PCR divided the 54 human symptomatic isolates of *Blastocystis* into ST1 (10/54, 18.5%) ST3 (29/54, 54.7%) and ST4 isolates (15/54, 27.8%) (Table 2). Then, PCR/HRM discriminated 29 ST3 isolates into wild (17/54, 31.5%), mutant (5/54, 9.3%), and heterozygous (7/54, 12.9%) intrasubtypes. No genetic variants were found for the ST1 and ST4 isolates. Our finding is supported by a previous study performed in Italy, where three distinct haplotypes (H1, H3 & H9) were detected among ST3 isolates obtained from Italian patients, and H1 was the most common with 34% [40]. Similarly, ST3 was segregated into Clade 1 (human infection) and Clades 2–4 (zoonotic infection) by multilocus sequencing [64]. In addition, only 3 haplotypes of ST3 were detected among human isolates collected from one area in rural central Mexico [58]. On the other hand, in a study performed in Iran using the barcoding region of the SSU rRNA gene, ST3 presented the highest allele variation in comparison to other STs [57]. However, ST3 showed 15 haplotypes in several areas in rural central Mexico [58]. Moreover, 38 genetic clusters of ST3 were obtained from a single French patient [43]. Regarding PCR/HRM, 17 *Blastocystis* STs were identified according to specific melting temperatures; however, no inter- or intrasubtypes were reported by sequencing [47], providing no genetic variations**.** In contrast, ST1-ST4 gave wild, mutant, and heterozygous variants after PCR/HRM curve analysis in other research [70]. Although our study showed a negative genetic variation among ST1 isolates, other studies demonstrated two intrasubtypes [53, 55]. Moreover, high ST1 genetic diversity was shown among Italian patients [9]. In agreement with our results related to the absence of genetic ST4 variants, two studies emphasized the homogeneity of this ST [40, 64]. In contrast, ST4 had 6 sequence clusters in another work [41]. The difference between our results and the results of others may be due to differences in geographical areas from which the isolates were collected, cultural behaviors temperature, genotyping method, and exposure to reservoir hosts [65].

Concerning polyp formation, in our work, all STs/intrasubtypes were shared in 44.5% of the lesions, whereas ST1 constituted 14.7%, wild, mutant, and heterozygous ST3 induced polyps in 12.9%, 3.8%, and 5.5%, respectively, and only 1.9% were related to ST4 (Table 2). Some previous studies support our findings regarding the outcome of *Blastocystis* infection and genetic variations, as *Blastocystis* isolated from persons affected or not with diarrhea showed 99.78% ST3 intrasubtype similarity [5]. Additionally, Vargas–Sanchez *et al***.** noticed that the presence of symptoms was not related to the genetic ST variants analyzed by PCR/HRM curves [68]. In contrast, *Blastocystis* exhibited intrasubtype variability in enteroadhesion, causing epithelial barrier dysfunction [73]. An explanation these conflicting data may be due to differences in host factors such as infective dose, diet, intestinal microbiota, oxidative stress, immunity, and intrasubtype virulence elements that could be reflected in *Blastocystis* infection outcomes [20].

In the present study, MUC2 immunostaining showed that 24/54 (44.5%) of the isolates gave a weak staining reaction (Table 3). ST1 constituted 14.7% (all induced polyps), wild ST3 totalled 16.7% (12.9% induced polyps), mutant ST3 represented 3.8% (all induced polyps), heterozygous ST3 composed 7.4% (5.5% induced polyps) and ST4 was responsible for 1.9% (induced polyps). In agreement with our results, *Blastocystis* degrades mucin from all segments of the large intestine [3]. Subsequently, MUC2 deficiency may lead to chronicity [69]. On the other hand, MUC2-negative mice showed higher cell proliferation and a significant decrease in apoptosis [72]. This increase in the ratio of proliferating to apoptotic cells is commonly associated with a precancerous colon [36]. Therefore, MUC2 deficiency may explain the presence of precancerous polyps among humans infected with *Blastocystis* [18] and experimentally infected rats [28]. In the HCT116 cell line, *Blastocystis* antigens extracted from ST1 symptomatic isolates increased the proliferation of cells and inhibited apoptosis [33]. In contrast, the upregulation of nuclear factor kappa light chain enhancer of activated B cells (NF-κB) in HCT116 cells exposed to symptomatic *Blastocystis* antigens led to the upregulation of MUC2 transcription [14]. Moreover, weak immunostaining of MUC2 was detected in 63.3% of patients with early CRC [6]. Interestingly, the loss of MUC2 expression was accompanied by a decrease in goblet cell numbers [45]. This explains our results related to goblet cell count, as all STs polyp inducers gave the lowest counts, particularly ST1 and wild ST3. These data are in agreement with Yakoob *et al*., who noticed that *Blastocystis* infection with ST1 and ST3 induced 33% and 60% goblet cell depletion, respectively [75]. This conflict may be explained by the fact that when goblet cells are chronically exposed to mucus pathogens, there is an increase in MUC2 secretion, leading to stress and goblet cell apoptosis, particularly in the case of prolonged stress [68].

In the current study, IELs were activated to varying degrees in terms of genetic variation and polyp formation with moderate infiltration in both the ST1 and ST3 wild intrasubtype (Table 4). In agreement with our results, Yakoob *et al*. showed that *Blastocystis* infection with ST1 and ST3 induced high numbers of IELs (43% and 20%, respectively) in the colon, leading to a proinflammatory response [74]. These results matched those of Pavanelli *et al*., who demonstrated that to fight *Blastocystis* infection, hyperplastic lymphoid aggregates increased through the synthesis and secretion of proangiogenic factors [50]. Moreover, cytotoxic T cells mediate an antiangiogenic effect by releasing more IFN-γ [12]. Subsequently, tumor growth is angiogenic-dependent [54]; therefore, in the case that IELs fail to suppress this angiogenic mechanism, excessive cytotoxicity induces epithelial cell damage and enhances polyp formation [18]. Moreover, ST1 modified 10 genes in the colon cancer cell line HC-29, which promoted cancer growth by stimulating angiogenesis [36, 37]. Additionally, patients infected with ST3 have a high level of IL-6 [7], which is an angiogenetic factor [56]. Moreover, ST3 induced the dysregulation of IFN-γ [34]. This may explain why the highest level of IELs were found among polypoid colons in the present study, whereas the lowest polyp formation by ST4 may be due to its downregulation of TNF-α [52].

In the present study, the numbers of mast cells regarding ST genetic variation and polyp formation (Table 4). An increase in IL-8 (an angiogenic factor) occurred in HT-29 cells exposed to ST1 by activating mast cells and releasing NF-κB, supporting our results [74]. Interestingly, NF-κB promotes nuclear entry of β-catenin during inflammation, leading to tumor initiation and formation [25]. Similarly, activated mast cells exposed to ST3 antigens induced higher upregulation of transforming growth factor beta (TGF-β) and IL-8 [34], which suppress the protective immune response in the tumor environment [71]. In contrast, some authors consider mast cells to exhibit antitumor activity by releasing IL-9 and heparin [76]. This may explain the insignificant results obtained related to ST1. However, the suppressor role that mast cells play in polyp formation in blastocystosis is still unclear.

In the current study, all ST-induced polyps showed invasion (100%), while only two isolates from the nonpolyp inducer (one samples from ST1 and wild ST3 intrasubtype) were invasive (8.6%). These findings have been demonstrated in mice with acute infection [22, 78]. Such invasive features of *Blastocystis* can indicate parasite virulence since tissue infiltration usually triggers more intense inflammatory reactions depending on the length of infection [50]. In contrast, no invasive forms were observed in rats after 4 weeks of infection with ST3 and ST4 [19]. In our study, the duration of experimental infection was 8 weeks; therefore, patients with severe prolonged infection were more susceptible to chronic inflammation and polyp formation than patients with the acute form.

In this study, the sIgA optical density of each ST/intrasubtype proved that the levels of sIgA among rats infected with ST1 and ST3 intersubtype-induced polyps were less than those in infected rats without polyps, and the difference was statistically significant (Table 5). These data were in agreement with the degradation effect of proteases secreted from invading *Blastocystis* that explain the low levels of sIgA [51]. In contrast, a significantly high sIgA level was linked to the presence of GIT symptoms [39]. Moreover, the immunogenic heterogeneity occurring with ST3 symptomatic isolates may play a role [62]. Although elevated fecal IgA is used as a potential marker for the early diagnosis of CRC [13] in chronic *Blastocystis* infection, low IgA levels may be a role of cancer predisposition, as shown by multiple studies [38].

In conclusion, our study hypothesized that *Blastocystis* infection possesses a potential carcinogenic effect that influences the abnormal growth of colorectal cells through an immune response. The knowledge and understanding of the association between *Blastocystis* infection, particularly STs, and colorectal cancer may provide insight into the prevention and/or development of new immune therapeutic strategies to combat colorectal cancer.
